# Biomimetically Reinforced Polyvinyl Alcohol-Based Hybrid Scaffolds for Cartilage Tissue Engineering

**DOI:** 10.3390/polym9120655

**Published:** 2017-11-28

**Authors:** Hwan D. Kim, Yunsup Lee, Yunhye Kim, Yongsung Hwang, Nathaniel S. Hwang

**Affiliations:** 1 School of Chemical and Biological Engineering, the Institute of Chemical Processes, Seoul National University, Seoul 08826, Korea; hwankim@snu.ac.kr (H.D.K.); ddub-_-z@hanmail.net (Y.L.); 2 Soonchunhyang Institute of Medi-Bio Science (SIMS), Soonchunhyang University, Cheonan-si, Chungcheongnam-do 31151, Korea; kyhye4@naver.com; 3 Institute of Tissue Regeneration, College of Medicine, Soonchunhyang University, Cheonan-si, Chungcheongnam-do 31151, Korea; 4 The BioMax Institute of Seoul National University, Seoul 08826, Korea

**Keywords:** polyvinyl alcohol (PVA), hyaluronic acid (HA), chondroitin sulfate (CS), chondrocyte, hydrogel, cartilage tissue engineering

## Abstract

Articular cartilage has a very limited regeneration capacity. Therefore, injury or degeneration of articular cartilage results in an inferior mechanical stability, load-bearing capacity, and lubrication capability. Here, we developed a biomimetic scaffold consisting of macroporous polyvinyl alcohol (PVA) sponges as a platform material for the incorporation of cell-embedded photocrosslinkable poly(ethylene glycol) diacrylate (PEGDA), PEGDA-methacrylated chondroitin sulfate (PEGDA-MeCS; PCS), or PEGDA-methacrylated hyaluronic acid (PEGDA-MeHA; PHA) within its pores to improve in vitro chondrocyte functions and subsequent in vivo ectopic cartilage tissue formation. Our findings demonstrated that chondrocytes encapsulated in PCS or PHA and loaded into macroporous PVA hybrid scaffolds maintained their physiological phenotypes during in vitro culture, as shown by the upregulation of various chondrogenic genes. Further, the cell-secreted extracellular matrix (ECM) improved the mechanical properties of the PVA-PCS and PVA-PHA hybrid scaffolds by 83.30% and 73.76%, respectively, compared to their acellular counterparts. After subcutaneous transplantation in vivo, chondrocytes on both PVA-PCS and PVA-PHA hybrid scaffolds significantly promoted ectopic cartilage tissue formation, which was confirmed by detecting cells positively stained with Safranin-O and for type II collagen. Consequently, the mechanical properties of the hybrid scaffolds were biomimetically reinforced by 80.53% and 210.74%, respectively, compared to their acellular counterparts. By enabling the recapitulation of biomimetically relevant structural and functional properties of articular cartilage and the regulation of in vivo mechanical reinforcement mediated by cell–matrix interactions, this biomimetic material offers an opportunity to control the desired mechanical properties of cell-laden scaffolds for cartilage tissue regeneration.

## 1. Introduction

Owing to the biomechanical, biochemical, and structural properties of the native extracellular matrix (ECM) of articular cartilage and its dynamic regulation by specialized cells, i.e., chondrocytes, articular cartilage exhibits mechanical stability against friction and wear and provides a load-bearing capability during joint movement [1,2]. However, due to its intrinsic avascular nature, articular cartilage has a limited regenerative and self-healing capacity, and, therefore, there has been a tremendous interest in the development of efficient tissue engineering-based strategies to treat cartilage defects, which include the use of stem cells, soluble growth factors, and scaffolds [3,4,5,6,7]. The therapeutic potential of stem cells and various soluble growth factors has been investigated extensively, but emerging evidence also suggests that recapitulating the physicochemical cues for articular cartilage with natural and synthetic polymer-based biomimetic materials could play an equally significant role in cartilage tissue homeostasis and regeneration [8,9,10]. 

Given the poor mechanical characteristics of native ECMs, despite their excellent biological and biochemical properties [11], recent advancements in the field of biomaterials have led to the development of mechanically robust hydrogel-based three-dimensional scaffolds by controlling their physicochemical properties, such as pore size, porosity, and matrix rigidity [12]. In particular, polyvinyl alcohol (PVA), which is the hydrolyzed form of polyvinyl acetate and an FDA-approved material, is widely used in various biomedical applications, including osteochondral grafts, artificial blood vessels, contact lenses, surgical sponges, and implantable medical devices [13,14]. Although PVA possesses desirable properties such as biocompatibility, nondegradability, low protein absorption, and easily tunable mechanical properties, it does not efficiently support cell adhesion on its surface owing to the hydrophilic moieties provided by the hydroxyl group (–OH) on its backbone [15,16]. 

To develop synthetic polymer-based scaffolds conferring better biomimetic microenvironments, a number of studies have investigated the effects of ECM components on tissue formation [17]. These approaches include various chemical modifications of cartilage ECM-derived components and their incorporation into synthetic polymers with tunable physicochemical properties to maintain chondrocyte phenotypes via the modulation of cell–cell and cell–matrix interactions [18,19,20]. Chondroitin sulfate (CS) and hyaluronic acid (HA) are the most abundant glycosaminoglycans (GAGs) in articular cartilage, and their abilities to provide biomechanical and physicochemical cues to embedded chondrocytes are well established [21,22]. 

Therefore, in the present study, we developed a biomimetic PVA-based hybrid scaffold that served dual functionalities as a biomechanically reinforced and a biochemically cartilage-mimicking scaffold. We further evaluated its in vitro functions and in vivo chondrogenic potential using an ectopic mouse model. The scaffold was successfully generated using a stepwise method. First, we employed macroporous PVA sponges as a base structure to provide elastic reinforcement and three-dimensional, interconnected pore spaces to allow mass transfer and cell infiltration. Second, we harnessed photocrosslinkable cartilage-specific bioactive components, including CS or HA, to photoencapsulate rabbit chondrocytes within the aforementioned pore structures. 

## 2. Materials and Methods

### 2.1. Materials

All chemicals and reagents were purchased from the vendors mentioned. The PVA sponge constructs were purchased from Medtronic Xomed Inc. (Merocel^®^; Jacksonville, FL, USA) and poly(ethylene glycol) diacrylate (PEGDA; *M*_W_ = 3400 Da) was purchased from Alfa Aesar (Haverhill, MA, USA). Sodium hyaluronate (HA; *M*_W_ = 1,600,000 Da) was obtained from Lifecore Co. (Chaska, MN, USA) and chondroitin sulfate (*M*_W_ = ~20,000–40,000 Da) was purchased from Tokyo Chemical Industry (Tokyo, Japan). Glycidyl methacrylate (GMA) and deuterium oxide were purchased from Sigma-Aldrich (St. Louis, MO, USA). Both Live–Dead Cell Viability–Cytotoxicity kits for evaluating cell viability and the Quanti-iT™ PicoGreen dsDNA Assay kit were obtained from Molecular Probes (Eugene, OR, USA). Papain and collagenase type II were obtained from Worthington Biochemical Corporation (Lakewood, NJ, USA) and the photoinitiator (Irgacure 2959) was purchased from Ciba Specialty Chemicals Inc. (Basel, Switzerland). 

### 2.2. Methacrylation of Chondroitin Sulfate and Hyaluronic Acid

Methacrylated chondroitin sulfate (MeCS) and hyaluronic acid (MeHA) were prepared by reacting the hydroxyl functional groups of both CS and HA with GMA, as described previously [22]. Briefly, CS was dissolved in phosphate-buffered saline (PBS) at a final concentration of 10% *w*/*v* and 10% *v*/*v* GMA was added dropwise to the mixture. Next, the reaction mixture was allowed to react for 11 days under vigorous stirring. The resulting solution was dialyzed in deionized (DI) water for 2 days using a dialysis membrane (MCO, ~1000 Da) and then lyophilized. Similarly, for MeHA, HA was dissolved in PBS at a final concentration of 1% *w*/*v*, and 2% *v*/*v* GMA was added dropwise. The reaction mixture was allowed to react for 8 days under vigorous stirring, dialyzed, and lyophilized. The ^1^H nuclear magnetic resonance (NMR) spectra for MeCS and MeHA were obtained using a Bruker 400 MHz spectrometer (Supplementary Figure S1 in Supplementary Materials). Both MeCS and MeHA were stored at −20 °C until future use.

### 2.3. Swelling Ratio Measurement and Mechanical Testing

The swelling ratio of each sample was determined using a gravimetric method. Briefly, hydrogels were swollen in PBS for 24 h to reach an equilibrium. The wet weights of the samples were measured after removing excess PBS from the surface of the hydrogels using a wet tissue paper. The weighed hydrogels were lyophilized to measure their dried weights. The swelling ratios of the hydrogels were determined according to the following equation:

$$\mathrm{Swelling}\;\mathrm{Ratio}\;\left(Q\right)=\;\frac{wet\;weight\;of\;equilibrated\;hydrogel\;in\;PBS}{dried\;weight\;of\;hydrogel\;after\;lyophilization}$$

To calculate Young’s modulus, hydrogels were swollen in PBS for 24 h to reach equilibrium swelling. Instron 5966 (Instron Corporation, Norwood, MA, USA) equipped with a 100 N load cell was used to measure the Young’s modulus of the samples. Prior to the compression test, the diameter and height of each hydrogel were gauged, and the samples were compressed up to 40% strain, at a strain rate of 1.5 mm/min. Young’s modulus was obtained from the linear region of the stress–strain curve (0–10% strain).

### 2.4. Isolation of Rabbit Chondrocytes and Cell Culture

Rabbit chondrocytes were isolated and cultured as described elsewhere [23]. Briefly, articular cartilage was dissected from New Zealand white rabbits (Koatech Laboratory Animal Company, Pyeongtaek, Korea). The cartilage pieces were washed with PBS three times and digested with 0.2% *w*/*v* collagenase type II (Worthington Biochemical Corporation, Lakewood, NJ, USA) in Dulbecco’s modified Eagle’s medium (DMEM) supplemented with 100 units/mL of penicillin/streptomycin for 18 h at 37 °C in 5% CO_2_. The isolated chondrocytes were washed and cultured in DMEM containing 10% *v*/*v* fetal bovine serum, 100 units/mL penicillin/streptomycin, 1% *v*/*v* 4-(2-hydroxyethyl)-1-piperazaineethanesulfonic acid (HEPES), 1% *v*/*v* non-essential amino acid (NEAA), 0.2% l-proline, and 0.2% l-ascorbic acid at 37 °C in 5% CO_2_. The medium was changed every 2 days.

### 2.5. Photoencapsulation of Chondrocytes in Cartilage-Specific Bioactive Components and Fabrication of Cell-Laden PVA-Based Hybrid Scaffolds

PVA-based hybrid scaffolds were created and their internal pores were filled with photopolymerized cell-laden hydrogels, such as photocrosslinkable PEGDA-MeCS, PEGDA-MeHA, or PEGDA. As a control group, a PVA-based hybrid scaffold was fabricated with PEGDA alone at a final concentration of 10% *w*/*v*. Hereafter, we refer to this scaffold as PVA-PEG (PVA + 10% *w*/*v* PEGDA). For the experimental groups, either 20% *w*/*v* MeCS or 2% *w*/*v* MeHA was prepared in PBS, mixed at a 1:1 ratio with 20% *w*/*v* PEGDA, and used to create PVA-based hybrid scaffolds, hereafter termed PVA-PCS (PVA + 10% *w*/*v* PEGDA + 10% *w*/*v* MeCS) and PVA-PHA (PVA + 10% *w*/*v* PEGDA + 1% *w*/*v* MeHA). Prior to cell seeding onto the PVA-based scaffolds, the PVA sponges were sterilized under UV light overnight. Finally, rabbit chondrocytes were mixed with PVA-PEG, PVA-PCS, or PVA-PHA precursor solutions at a concentration of 1 × 10^6^ cells/construct in the presence of a photoinitiator (Irgacure 2595) (final concentration, 0.05% *w*/*v*) and slowly injected into the PVA sponges. After the precursor solutions, containing rabbit chondrocytes, filled the internal pores of the PVA sponges, the constructs were exposed to UV light (3.5 mW/cm^2^) for 5 min. The constructs were removed from the mold and cultured at 37 °C in 5% CO_2_ in chondrocyte growth medium. The culture medium was changed every 2 days for 3 weeks. As a control seeding method, rabbit chondrocytes were directly seeded onto the PVA sponge without adding the photopolymerizable hydrogels. 

### 2.6. Cell Viability

To determine cell viability after 24 h of photoencapsulation within PVA-based hybrid scaffolds, a live–dead assay was performed using the Live–Dead Cell Viability–Cytotoxicity kit (L-3224; Molecular Probes). Briefly, the cell-laden scaffolds were vertically cut into thin slices and incubated with calcein-acetoxymethyl ester (AM) for labelling live cells and ethidium homodimer-1 (EthD-1) for labelling dead cells. Multiple random images were obtained using the Zeiss 720 confocal laser scanning microscope, and cell viability was subsequently evaluated by counting the number of viable and dead cells using ImageJ (NIH, Bethesda, MD, USA).

### 2.7. Biochemical Assays

Biochemical assays were performed using cell-laden PVA-ECM hybrid scaffolds. The samples were collected after 24 h for evaluating the initial cell retention. The samples were also collected after 3 weeks of in vitro culture, lyophilized for 24 h, and digested using 1 mL of papain solution (125 µg/mL) for 16 h at 60 °C, as described previously [24]. The DNA content was quantified using the Quanti-iT™ PicoGreen dsDNA Assay kit. The amount of glycosaminoglycan (GAG) was measured by the dimethylmethylene blue spectrophotometric assay at A_525_, as described previously [25]. The collagen content was determined by measuring the amount of hydroxyproline within the constructs after acid hydrolysis at 115 °C for 18 h, followed by reaction with *p*-dimethylaminobenzaldehyde and chloramine-T, as reported previously [26]. The amount of DNA was normalized to the dried weight of the constructs, and both GAG and collagen contents were also normalized to the corresponding DNA content.

### 2.8. Quantitative PCR (qPCR)

Cell-laden scaffolds were analyzed by qPCR after 3 weeks of in vitro culture. Total RNA was extracted from the samples using TRIzol (Life Technologies, Carlsbad, CA, USA), and reverse transcription was performed using the M-MLV cDNA Synthesis kit (Enzynomics, Seoul, Korea), according to the manufacturer’s instructions. Quantitative PCR was performed using SYBR Green PCR Mastermix (Life Technologies) on ABI Step One Plus™ Real-Time PCR System (Applied Biosystems, Foster City, CA, USA). The expression levels of the genes of interest were normalized against *GAPDH* levels as a reference, and ΔCt values were determined as follows: Ct^target^ − Ct^GAPDH^. The relative fold changes were calculated as 2^−^^ΔΔCt^, as described previously [27]. The PCR primers used in the present study are listed in Supplementary Table S1 (Supplementary Materials). 

### 2.9. Animal Studies

All in vivo experimental procedures were performed in accordance with the Guide for the Care and Use of Laboratory Animals by the Seoul National University. The cell-laden PVA-ECM hybrid scaffolds were preconditioned in chondrocyte growth medium for 3 days in vitro prior to transplantation. For subcutaneous implantation of the cell-laden scaffolds (5 mm diameter; 2.75 mm, height) into the subcutaneous pouches (left or right cranial and caudal), 4-week-old BALB/c nude mice (16–20 g; Charles River, Wilmington, MA, USA) were anesthetized by the administration of ketamine (100 mg/kg) and xylazine (10 mg/kg), and then a small incision (less than 1 cm) was made in the back of each mouse. After the surgery, all mice were housed in separated cages for 6 weeks. New cartilage tissue formation in vivo was evaluated histologically, and the mechanical reinforcement by cell-secreted ECMs was determined by compression tests. 

### 2.10. Histological Analysis

Cell-laden scaffolds were fixed with 4% paraformaldehyde solution at 4 °C overnight. Prior to embedding in optimal temperature cutting compound (OCT), the samples were incubated in a 20% sucrose solution for 2 h at room temperature, and the OCT-embedded samples were placed in isopentane and frozen in liquid nitrogen. Using a cryostat (CM3050; Leica, Wetzlar, Germany), the samples were cryosectioned into 15 µm-thick slices. For hematoxylin and eosin (H&E) staining, frozen sections were rehydrated in PBS at room temperature for 10 min, stained with hematoxylin (Gill 2, cat#: 3536-16; Ricca Chemical Company, Arlington, TX, USA) for 5 min, and thoroughly rinsed with DI water. The sections were subsequently stained with Eosin-Y (cat#: 7111; Richard-Allan Scientific, San Diego, CA, USA) for 1 min, followed by multiple washes in DI water. The rehydrated sections were stained with 0.1% Safranin-O (ScholAR Chemistry, Rochester, NY, USA) for 5 min at room temperature. The samples were gradually dehydrated in a graded series of ethanol, followed by CitriSolv (cat#: 22-143975; Fisher Scientific, Waltham, MA, USA). 

For performing the immunofluorescence staining of collagen type I, II, and F-actin, rehydrated sections were blocked and permeabilized in PBS containing 0.3% Triton X-100 and 3% bovine serum albumin (cat# A7906; Sigma) for 1 h at room temperature. Next, the sections were incubated with primary antibodies against type I and type II collagens (1:200; Abcam, Cambridge, UK) at 4 °C overnight. After washing in PBS, the sections were incubated with secondary antibodies (1:250; goat anti-mouse Alexa-Fluor 488 and goat anti-mouse Alexa-Fluor 546; Life Technologies) and Alexa-Fluor 488 Phalloidin (1:100; Life Technologies) to visualize F-actin. The nuclei were stained with Hoechst 33342 (2 μg/mL; Life Technologies) for 5 min at room temperature, and the sections were mounted with Canada balsam (Sigma-Aldrich) for further storage. Imaging was performed using a fluorescence microscope (CKX-41; Olympus, Tokyo, Japan).

### 2.11. Statistical Analysis

All data are presented as means ± standard deviation (SD). Differences between groups were evaluated using Student’s *t*-tests with significance thresholds of * *p* < 0.05, ** *p* < 0.01, and *** *p* < 0.005.

## 3. Results and Discussion

### 3.1. Development of Novel PVA-Based Hybrid Scaffolds with Photopolymerizable Cell-Laden Cartilage-Specific Bioactive Components

**Figure 1:**
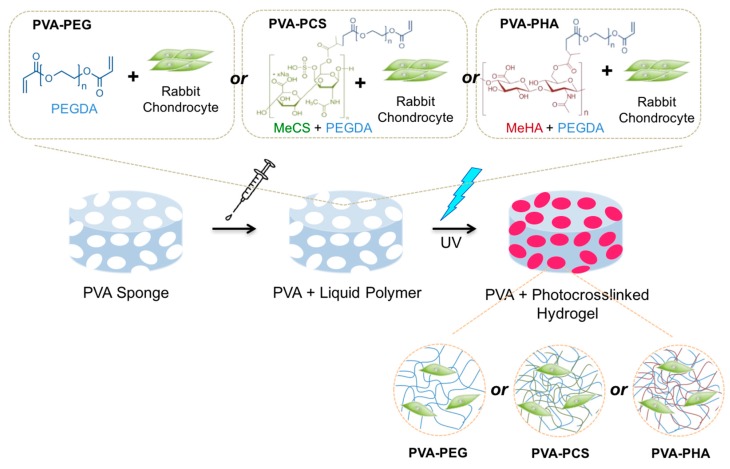
Schematic illustration of the stepwise method for embedding rabbit chondrocytes in photocrosslinkable PEGDA or PEGDA with cartilage-specific bioactive components (MeCS or MeHA), within macroporous PVA-based scaffolds. Abbreviations for PEGDA: poly(ethylene glycol) diacrylate; MeCS: methacrylated chondroitin sulfate; MeHA: methacrylated hyaluronic acid; PVA; polyvinyl alcohol.

Numerous studies have demonstrated the potential applications of PVA-based scaffolds in tissue engineering, including osteochondral tissue regeneration [7,15,28,29]. Hence, to engineer novel biomimetic materials for cartilage tissue repair, we employed a macroporous PVA sponge as a base material to recapitulate physiologically relevant structural and functional properties of the native cartilage tissue. A schematic illustration of the development of the chondrocyte-embedded PVA-based hybrid scaffolds is shown in Figure 1. In particular, the PVA sponge network provide three-dimensional and porous internal microstructures similar to those in native ECMs, such as collagen, which is abundant in cartilage tissue. Moreover, by utilizing photopolymerizable cartilage-specific bioactive components, such as MeCS and MeHA, for the encapsulation of primary rabbit chondrocytes within the pores of the PVA sponges, the functional properties of native cartilage were successfully recapitulated using both proteoglycans (cartilage-specific bioactive components) and chondrocytes (cellular components). This could be achieved by slowly filling the internal pores of the PVA sponges with photopolymerizable PEGDA-MeCS (PCS), PEGDA-MeHA (PHA), or PEGDA containing isolated chondrocytes, followed by UV photopolymerization.

**Figure 2:**
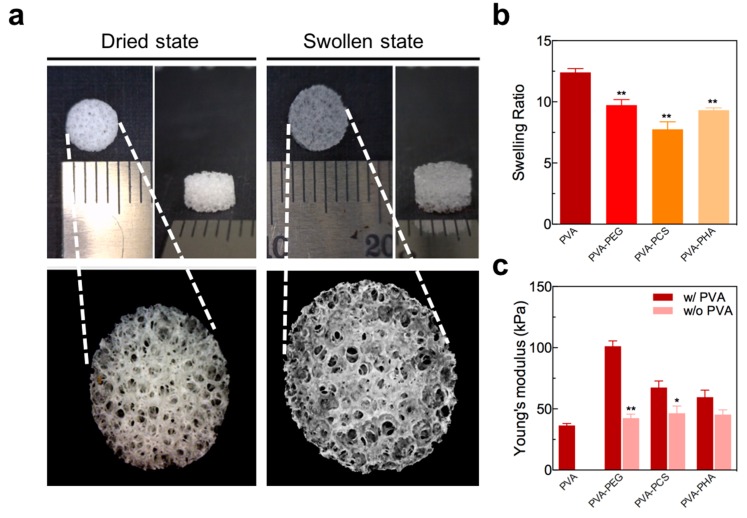
(**a**) Gross images of porous PVA sponges in dried (left) and swollen (right) states, (**b**) equilibrium swelling ratios of samples in PBS (PVA indicates a macroporous PVA scaffold without photopolymerized hydrogels), (**c**) Young’s modulus of the acellular PVA sponge alone (PVA), photocrosslinked hydrogels (shown in bright pink), and PVA-based hybrid scaffolds with combinations of the PVA sponge and photocrosslinked hydrogels (shown in dark red). Values represent the means ± SD. * *p* < 0.05 and ** *p* < 0.01.

As shown in Figure 2, the gross images of the PVA sponges in both dried and swollen states clearly indicated that the PVA sponges have highly porous and interconnected pore structures. Accordingly, the acellular PVA sponges alone had a high swelling ratio (~12.4 ± 0.44), whereas the incorporation of photopolymerizable PEGDA, PCS, or PHA into the PVA sponges resulted in lower swelling ratios, i.e., 9.73 ± 0.63, 7.75 ± 0.86, and 9.32 ± 0.27, respectively (Figure 2b). This might be explained by the higher degrees of crosslinking introduced by photopolymerizable PEGDA, PCS, or PHA compared to PVA alone. In addition to the increased crosslinking density of the networks, the significant decrease in the swelling ratio of the PVA sponges in the presence of photopolymerizable hydrogels might be explained by the physical occupation of pore spaces with PEGDA, PCS, or PHA hydrogels, thereby minimizing water retention within the network, which is in accordance with previous reports [30,31]. 

Next, we examined the compressive moduli of hydrated (equilibrated) acellular scaffolds to evaluate the contributions of various photopolymerizable cartilage-specific bioactive components to the mechanical reinforcement of the PVA-based scaffolds. In the absence of the PVA sponge, PEGDA, MeCS, and MeHA acellular hydrogels (indicated by bright pink bars in Figure 2c) showed Young’s moduli similar to that of the PVA sponge alone. However, these hydrogels showed a lower degree of elastic deformation than the PVA sponge, which exhibited elastic deformation up to a strain of 40% without any breakage (data not shown). Thus, considering the structure–function relationship of native articular cartilage [32], our novel approach to recapitulate the biomechanical characteristics of the cartilage tissue includes the incorporation of photocrosslinkable hydrogels (PEGDA, PCS, or PHA) within the internal pores of the PVA sponges, resulting in improved mechanical properties, such as matrix rigidity (Young’s modulus) and deformation without breakage (toughness), of the PVA-based hybrid scaffolds (represented as dark red bars in Figure 2c) as compared to those of their counterparts (photocrosslinkable hydrogels alone), as shown in Figure 2c (represented as bright pink bars in Figure 2c). These results clearly revealed that the introduction of photocrosslinkable moieties, such as PEGDA, PCS, and PHA, into the interconnected pores of PVA sponges, yielding hybrid structured scaffolds, reinforced the mechanical characteristics of the PVA-based acellular scaffolds, not only by increasing the crosslinking density throughout the scaffold via interpenetrating polymer network formation, but also by the presence of incompressible moieties (PEGDA, PCS, or PHA) within the pores, which biophysically mimic the functions of articular cartilage itself [2,30]. 

### 3.2. Effects of the Cartilage-Specific Bioactive Components on Cell Retention and Viability

**Figure 3:**
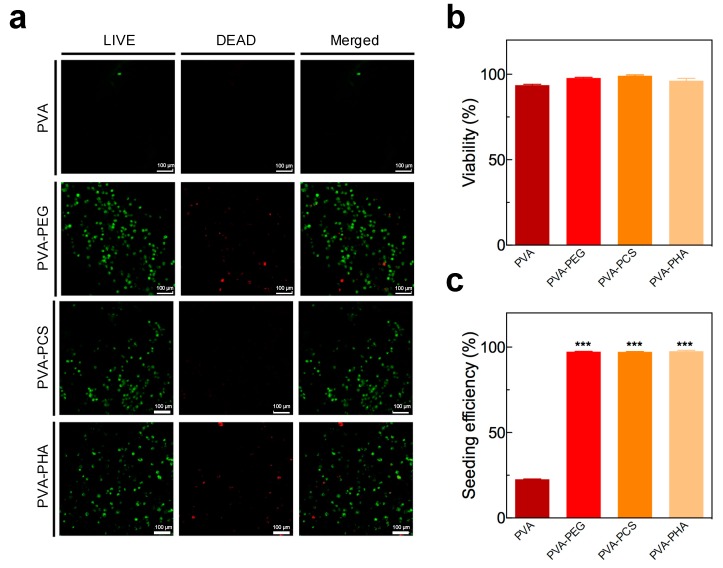
(**a**) Live–dead assay of directly seeded or photoencapsulated rabbit chondrocytes within PVA-based scaffolds. Viable cells were stained with Calcein-acetoxymethyl ester (AM) (shown in green) and dead cells were stained with ethidium homodimer-1 (Ethd-1) (shown in red). Scale bar = 50 μm. (**b**) Cell viability was calculated as the ratio of the number of viable cells to the total number of cells. (**c**) The seeding efficiency of directly seeded or photoencapsulated rabbit chondrocytes within PVA sponges was calculated as the ratio of the number of cells within the construct to the total number of cells. Values represent the means ± SD. *** *p* < 0.005.

To evaluate the effect of the cartilage-specific photopolymerizable moieties within macroporous PVA-based scaffolds on the initial cell retention and viability, we performed a live–dead analysis of isolated chondrocytes that were either directly seeded onto the PVA sponges without cell encapsulation or photoencapsulated in the internal pores of the PVA sponges using PEGDA, PCS, or PHA precursors. As shown in Figure 3a,b, after seeding for 24 h or photoencapsulating the cells within the scaffolds, we observed that the cells, regardless of the composition of the PVA-based scaffold, exhibited high viability (>90%), and there was no significant difference among experimental groups. However, more importantly, we detected a dramatic decrease in cell retention when the cells were seeded directly onto the PVA sponges without any support from photopolymerizable moieties (Figure 3a, top row). Cell retention or seeding efficiency were further quantified by comparing the amount of DNA from cells initially seeded into each scaffold and the actual amount of DNA that was measured from each scaffold after 24 h using DNA assays. Briefly, after 1 × 10^6^ cells were seeded onto each construct for 24 h, the PVA sponge alone exhibited a cellular retention of only 22.59%, whereas the other groups (PVA-PEG, PVA-PCS, and PVA-PHA) exhibited 97.13%, 97.26%, and 97.67% seeding efficiencies, respectively (shown in Figure 3c). Poor cell retention within the PVA sponge could be attributed to its pore architecture. Sobral et al. [33] previously established that the architecture of scaffolds could affect the initial cell adhesion and seeding efficiency. For example, they revealed that scaffolds with large pore sizes and open pore structures typically show low cell retention within scaffolds. Thus, these results indicated that the introduction of photopolymerizable hydrogel moieties throughout the interconnected open pores within the PVA sponges, as a form of physical confinement, substantially improves the initial cell retention within the PVA-based hybrid scaffolds. 

### 3.3. Cell-Secreted ECM-Driven Mechanical Reinforcement of PVA-Based Hybrid Scaffolds

**Figure 4:**
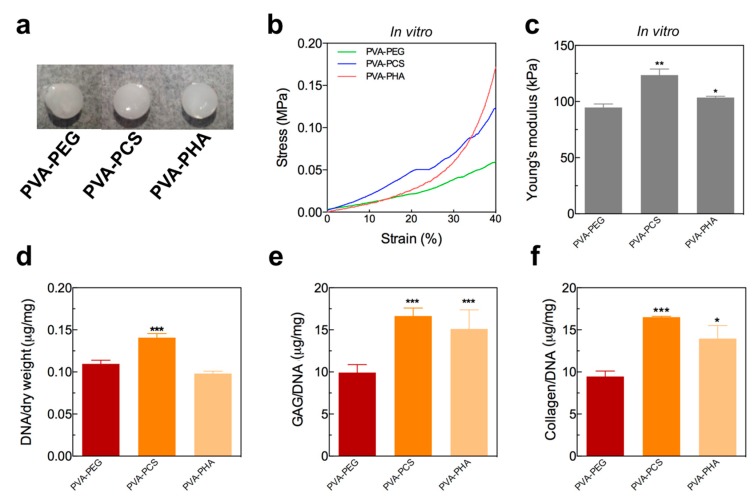
(**a**) Gross image of PVA-PEG, PVA-PCS, and PVA-PHA hybrid scaffolds, (**b**) stress–strain curves of the cellular PVA-based hybrid scaffolds after 3 weeks of in vitro culture, (**c**) Young’s modulus of the cellular PVA-based hybrid scaffolds after 3 weeks of in vitro culture, (**d**) quantification of the amount of DNA and (**e**,**f**) cartilage-specific ECM accumulation (GAG and total collagen) by chondrocytes. Values represent the means ± SD. * *p* < 0.05, ** *p* < 0.01, and *** *p* < 0.005.

Next, we examined the effect of the cartilage-specific bioactive moieties on cellular functions of the photoencapsulated chondrocytes within PVA-ECM hybrid scaffolds. After 3 weeks of in vitro culture, the gross images of the PVA-based scaffolds (PVA-PCS and PVA-PHA) demonstrated that PVA-PCS and PVA-PHA hybrid scaffolds could promote cell-secreted ECM deposition throughout the scaffolds (Figure 4a). To validate our observations, we performed mechanical testing to assess whether cell-secreted ECMs could induce the mechanical reinforcement of the cell-laden, PVA-based hybrid scaffolds. Based on the stress–strain curves shown in Figure 4b,c, we calculated Young’s moduli of the PVA-based hybrid scaffolds. Cellular PVA-PEG hybrid scaffolds did not show significant differences in mechanical strength compared to their acellular counterparts; however, the mechanical properties of both cellular PVA-PCS and PVA-PHA scaffolds were significantly enhanced by 83.30% and 73.76%, respectively, compared to those of the control groups (acellular PVA-PCS and PVA-PHA). Furthermore, we observed that the mechanical properties, including toughness that is defined as the area under the stress–strain curve, of the PVA-PHA scaffolds were significantly enhanced, which was possibly due to the fact that the HA used in the current study had a higher molecular weight (~1.6 × 10^6^ Da) than CS (~20–40 × 10^3^ Da) and PEGDA (~3400 Da). Thus, our results suggested that cells embedded within PVA-PCS or PVA-PHA may produce higher amounts of cartilage-specific ECMs compared to their counterparts. 

To validate our hypothesis that these mechanical reinforcements could be achieved by cartilage-specific ECMs deposited by the encapsulated cells within these biomimetic PVA-PCS and PVA-PHA hybrid structures, we performed biochemical analyses of cells grown on PVA-based scaffolds for 3 weeks in vitro. Similar to the mechanical reinforcement on the PVA-PCS and PVA-PHA hybrid scaffolds, biochemical analyses also showed that cells encapsulated in the PVA-PCS scaffolds were characterized by increased proliferation compared to cells encapsulated in PVA-PEG or PVA-PHA. More importantly, the amounts of accumulated cartilage-specific ECMs, including GAG and collagen, on both the PVA-PCS and the PVA-PHA scaffolds were significantly higher than those on their counterpart, PVA-PEG (Figure 4d–f). Overall, the hybrid structures generated by the incorporation of cartilage-specific bioactive components (both CS and HA) on the PVA sponge were beneficial for GAG and collagen accumulation, whereas the introduction of the same hybrid structure in the absence of a cartilage-specific bioactive component (PVA-PEG) could not improve GAG and collagen accumulation, proving that the presence of cartilage-specific bioactive components might be required to maintain the normal physiological phenotypes of encapsulated chondrocytes. Furthermore, these bioactive cartilage-specific bioactive components that photoencapsulated chondrocytes could strengthen the mechanical properties of the PVA-ECM hybrid scaffolds by progressive accumulation of cell-secreted ECMs. This is in agreement with previous studies demonstrating that the mechanical properties of engineered cartilage tissues improve substantially as a function of culture time, and this could be achieved through the accumulation of GAG and collagen synthesized by the embedded cells [34,35]. 

### 3.4. Gene Expression Analysis of Encapsulated Chondrocytes in PVA-Based Hybrid Scaffolds

**Figure 5:**
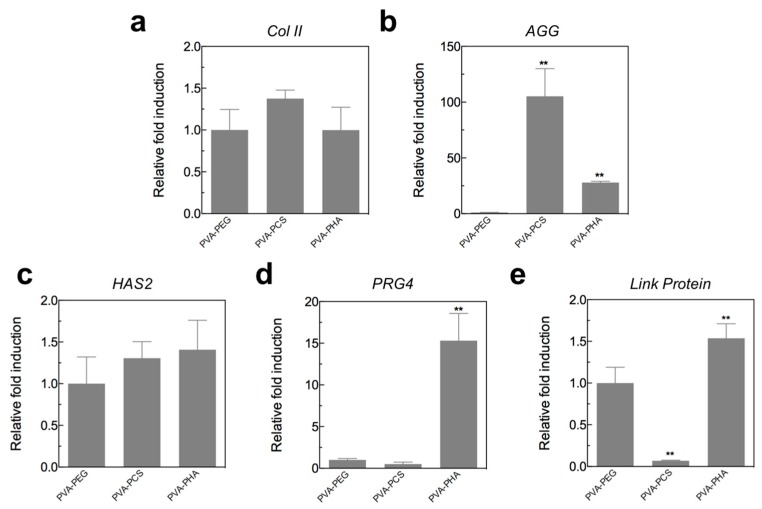
Quantitative PCR analysis of cartilage function-related genes, i.e., (**a**) Type II collagen (*Col II*), (**b**) Aggrecan (*AGG*), (**c**) Hyaluronan Synthase 2 (*HAS2*), (**d**) Proteoglycan 4 (*PRG4*), and (**e**) *Link Protein*, after 3 weeks of in vitro culture. All gene expression levels were normalized to the levels observed in the control group (PVA-PEG); *GAPDH* was used as the reference gene. Values represented the means ± SD. ** *p* < 0.01.

To further corroborate our findings from mechanical and biochemical analyses, we investigated the gene expression levels of chondrocyte-specific markers, including type II collagen (*Col II*) and aggrecan (*AGG*), in cells cultured on all PVA-based hybrid scaffolds (PVA-PEG, PVA-PCS, and PVA-PHA). As shown in Figure 5a,b, the chondrogenic gene expression profiles of *Col II* and *AGG* revealed that there was a slight upregulation of *Col II* in cells encapsulated within the PVA-PCS scaffolds, but the expression levels did not differ significantly among groups. However, the levels of *AGG* in cells encapsulated within both the PVA-PCS and the PVA-PHA hybrid scaffolds were dramatically upregulated, consistent with the results of the mechanical and biochemical analyses (shown in Figure 4).

In addition, we evaluated the gene expression levels of other chondrocyte-related markers, such as proteoglycan 4 (*PRG4*), *Link Protein*, and hyaluronan synthase 2 (*HAS2*). PRG4, also known as lubricin, is a mucinous glycoprotein and is abundantly present both in the synovial fluid and at the superficial layer of articular cartilage [36,37]. In addition, numerous studies have demonstrated that it has major roles in maintaining the boundary lubrication properties of articular cartilage by reducing the friction coefficient of the cartilage surface; therefore, it could protect the gliding surface of articular cartilage against degradation [38,39,40,41,42]. Additionally, link protein and HAS2 are the major components of cartilage-specific ECMs, and their critical roles in stabilizing proteoglycan aggregates and hyaluronic acid as well as their synthesis have been described in detail elsewhere [43,44]. Thus, we investigated how these bioactive cartilage-specific components could affect the functions of the encapsulated chondrocytes. As shown in Figure 5c, we observed a slight upregulation of *HAS2* in cells cultured on both the PVA-PCS and the PVA-PHA hybrid scaffolds; however, no statistically significant differences were observed among groups. Next, despite the lack of significant differences in *PRG4* levels among cells cultured on both the PVA-PEG and the PVA-PCS hybrid scaffolds, the PVA-PHA hybrid scaffold could support dramatically upregulated levels of *PRG4* (>15-fold). Similar to *PRG4*, we observed that the expression of the *Link Protein* gene was also upregulated in cells cultured on the PVA-PHA hybrid scaffold and decreased dramatically in cells cultured on PVA-PCS. These findings suggested that utilizing CS moieties for encapsulated chondrocytes could strongly enhance GAG synthesis, whereas exploiting HA moieties for encapsulated chondrocytes could promote GAG, link protein, and lubricin synthesis. Taken together, these results indicated that layered or multilayered structures of articular cartilage could be potentially obtained by utilizing a specific bioactive component responsible for biomimetically engineering the zonal architecture and recapitulating the physicochemical properties of the native articular cartilage tissue. 

### 3.5. In Vivo Cartilage Tissue Formation and Cell-Secreted ECM-Based Mechanical Reinforcement of the PVA-Based Scaffold 

**Figure 6:**
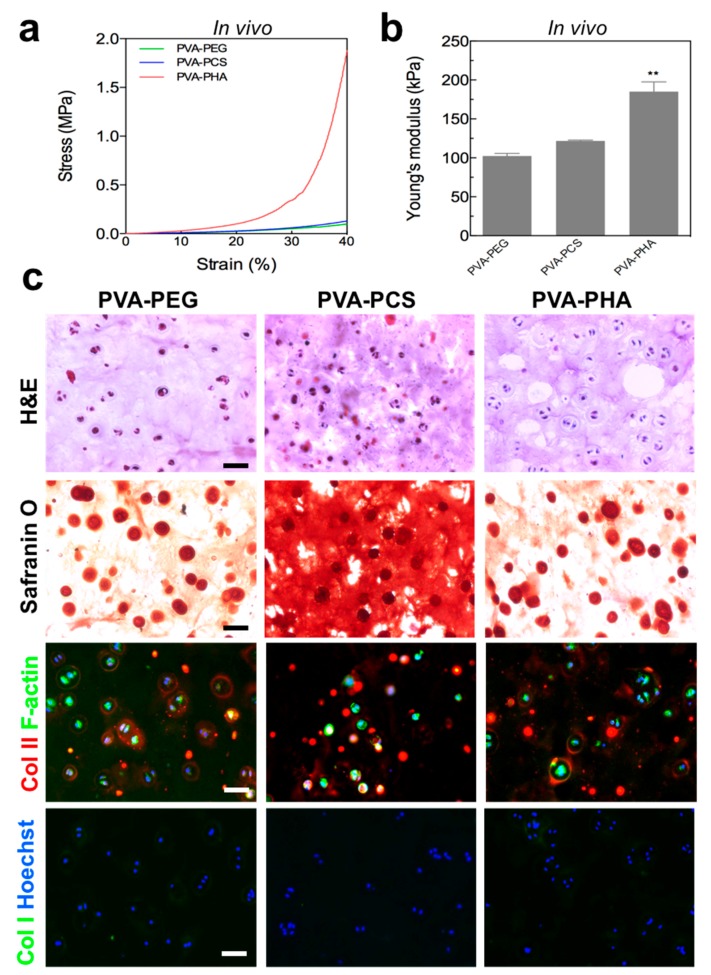
(**a**) Stress–strain curves and (**b**) Young’s modulus of cellular PVA-based hybrid scaffolds after 6 weeks of in vivo subcutaneous implantation, (**c**) cartilage-specific ECM accumulation visualized by hematoxylin and eosin (H&E) (1st row), Safranin-O (2nd row), type II collagen (3rd row), and type I collagen (4th row) immunofluorescence staining after 6 weeks of in vivo subcutaneous implantation. Scale bar = 50 μm. Values represent the means ± SD. ** *p* < 0.01.

Having demonstrated the in vitro chondrogenic potential of the PVA-PCS and the PVA-PHA hybrid scaffolds and the mechanical reinforcement mediated by the cell-secreted ECM, we subcutaneously transplanted the cell-laden PVA-based hybrid scaffolds into BALB/c nude mice and, after 6 weeks, we assessed the in vivo ectopic cartilage tissue formation mediated by the cartilage-specific bioactive components. Based on the stress–strain curves shown in Figure 6a,b, we evaluated the mechanical properties of cell-laden PVA-based scaffolds. Similar to the results obtained from in vitro mechanical testing, we observed that the compressive modulus for the cellular PVA-PEG scaffold collected from an ectopic mouse model showed no difference between acellular and in vitro cell-laden PVA-PEG scaffolds, demonstrating a lack of mechanical reinforcement. On the other hand, the compressive modulus of a PVA-PCS hybrid scaffold transplanted in vivo was approximately 121.74 kPa and was evidently augmented by 80.53% compared to that of its acellular counterpart, where the degree of mechanical reinforcement of the PVA-PCS scaffolds through in vivo ectopic cartilage tissue formation was equivalent to that of the in vitro cell-laden PVA-PCS hybrid scaffolds (~83.30%). Surprisingly, we found that the mechanical properties of the PVA-PHA hybrid scaffolds were remarkably strengthened (~185.20 kPa) through the in vivo ectopic cartilage tissue formation by 210.74% and 78.83% compared to those of their acellular and in vitro cell-laden PVA-PHA scaffolds, respectively. Although further studies are needed, this dramatic mechanical reinforcement of the PVA-PHA scaffolds that is mediated by ectopic cartilage tissue formation could be due to the presence of HA moieties within the scaffold and to their interaction with the host microenvironment. Despite its in vivo cell-matrix interaction-mediated mechanical reinforcement, its matrix rigidity was lower than that of the previously reported Young’s moduli of the superficial layer of rabbit articular cartilage, i.e., ~0.52 MPa [45]. Pioneered by Burdick and his coworkers, HA has been implicated in the regulation of cell signaling, matrix remodeling, and regeneration of various tissues [46,47]. In particular, HA is a major component of articular cartilage and has been shown to mediate the chondrogenic differentiation of stem cells through interactions with cell surface receptors such as CD44 and CD168 [19,48,49]. Thus, it is possible that endogenous stem cell populations within host tissues are able to interact with the HA moieties present within the PVA-PHA scaffolds, resulting in the accumulation of cartilage-specific ECMs and potentially contributing towards matrix stiffening. Moreover, numerous studies have demonstrated that HA possesses a limited immunogenicity due to its ability to minimize protein adsorption, and, therefore, HA could enhance in vivo cell viability following transplantation [50,51]. 

To verify whether the enhanced mechanical reinforcement observed by mechanical testing was indeed caused by ectopic cartilage tissue formation within host tissues, we performed histological evaluations of the in vivo transplanted cell-laden PVA-based scaffolds (Figure 6c). H&E and Safranin-O staining of the cell-laden scaffolds after 6 weeks from subcutaneous transplantation clearly demonstrated that the PVA-PCS and the PVA-PHA hybrid scaffolds showed increased lacunae formation surrounding round chondrocytes and ectopically deposited cartilage-specific ECMs throughout the scaffolds, without inflammatory responses, compared to the PVA-PEG scaffold; this is a characteristic physiological phenotype of articular chondrocytes [52]. Moreover, ectopic cartilage-specific tissue formation was further confirmed by the detection of type II collagen using immunofluorescence; the peripheral regions of the cells were positively stained for newly synthesized cartilage-specific ECM. In contrast, these cell-secreted ECMs did not stain for type I collagen, an indicator of dedifferentiated chondrocytes, demonstrating that the cartilage-specific bioactive ECM microenvironments were able to maintain the physiological phenotypes of articular chondrocytes following in vivo transplantation. Previously, we successfully demonstrated that harnessing cartilage-specific ECM components or integrin-binding peptides to provide embedded chondrocytes with favorable microenvironments could maintain the physiological phenotypes of chondrocytes during in vitro culture [21,22,53]. Furthermore, Wang et al. revealed that the ability to recapitulate the physiological functions of cartilage in vitro could lead to robust in vivo cartilage-specific ECM accumulation and tissue formation within the host [54,55]. Taken together, these results highlighted the importance of recapitulating the structures and functions of native cartilage tissues with cartilage-specific bioactive components in the regulation of both in vitro and in vivo chondrocyte functions and their corresponding contributions to the mechanical reinforcement of the PVA-PCS and the PVA-PHA hybrid scaffolds. 

## 4. Conclusions

In summary, the present study demonstrated that recapitulating physiologically relevant structural and functional properties of cartilage tissue by introducing cell-embedded photocrosslinkable MeCS or MeHA within the pores of PVA sponges could successfully promote in vitro cartilage-specific GAG and collagen accumulation within the scaffolds. Furthermore, photocrosslinkable MeCS- and MeHA-mediated in vitro and in vivo cartilage-specific tissue formation could result in the mechanical reinforcement of cell-laden PVA-based hybrid-structured scaffolds. The present study provides a proof of principle that biomimetically engineered zonal architectures of articular cartilage with desired mechanical properties can be achieved by utilizing MeCS or MeHA as dynamically tunable microenvironments. 

## Supplementary Materials

The following are available online at www.mdpi.com/2073-4360/9/12/655/s1, Figure S1: ^1^H spectra, Table S1: List of primers used for quantitative PCR.
