# Supplementary material for: Biomimetically Reinforced Polyvinyl Alcohol-Based Hybrid Scaffolds for Cartilage Tissue Engineering
Source: Polymers (Basel). 2017 Nov 28;9(12):655. doi: 10.3390/polym9120655 (PMC6418829; doi:10.3390/polym9120655)
Supplement: Supplementary file 1 [file polymers-09-00655-s001.pdf]

**Supplementary material**

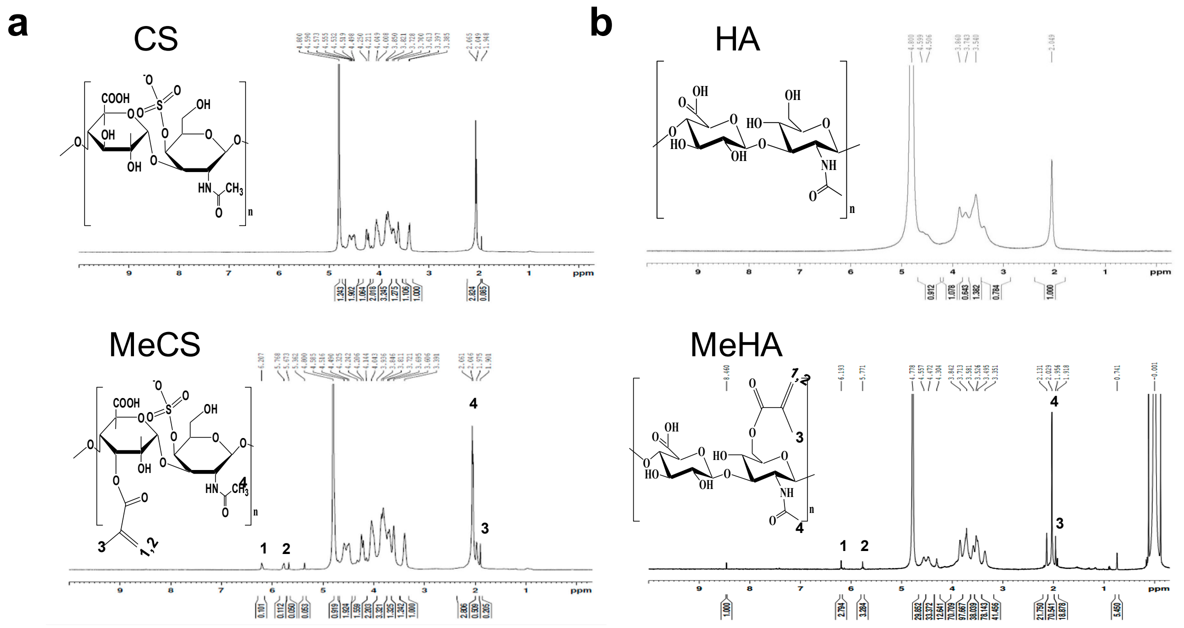


**Figure S1**. $^1$H-NMR spectra of (a) CS and MeCS with acrylate peaks at 5.3 ppm to 6.2 ppm indicating the presence of methacrylation of MeCS, (b) HA and MeHA with acrylate peaks at 5.7 ppm to 6.2 ppm indicating the presence of methacrylation of MeHA.

| Gene | Sequence (5' -3') |
|---|---|
| *GAPDH* | F: TCA CCA TCT TCC AGG AGC GA<br>R: CAC AAT GCC GAA GTG GTC GT |
| Type II collagen (*Col II*) | F: TTC ATG AAG ATG ACC GAC GA<br>R: GAC ACG GAG TAG CAC CAT CG |
| Aggrecan (*AGG*) | F: CCT TGG AGG TCG TGG TGA AAG G<br>R: AGG TGA ACT TCT CTG GCG ACG T |
| Proteoglycan 4 (*PRG4*) | F: TTA CCG ATG TCT GGG GCA TAC CTT C<br>R: TGG GCA GTG ATA TAG CTG AGG TGA CC |
| *Link Protein* | F: AGG CTG TAC AAC AGA GCA CC<br>R: AAA CAA GTC CCG GCT CTC AG |
| Hyaluronan Synthase 2 (*HAS2*) | F: TTT CTT TAT GTG ACT CAT CTG TCT CAC CGG<br>R: ATT GTT GGC TAC CAG TTT ATC CAA ACG G |

**Table S1.** List of primers used for quantitative PCR.
